# Double autoinhibition mechanism of signal transduction ATPases with numerous domains (STAND) with a tetratricopeptide repeat sensor

**DOI:** 10.1093/nar/gkz112

**Published:** 2019-02-21

**Authors:** María-Natalia Lisa, Virginija Cvirkaite-Krupovic, Evelyne Richet, Gwenaëlle André-Leroux, Pedro M Alzari, Ahmed Haouz, Olivier Danot

**Affiliations:** 1 Unité de Microbiologie Structurale, Institut Pasteur, CNRS UMR 3528 & Université Paris Diderot, 75724 Paris Cedex 15, France; 2 Instituto de Biología Molecular y Celular de Rosario (IBR, CONICET-UNR), Ocampo y Esmeralda, S2002LRK, Rosario, Argentina; 3 Unité de Génétique moléculaire, Institut Pasteur, CNRS ERL 3526, 75724 Paris Cedex 15, France; 4 Unité de Biologie moléculaire du gène chez les extrêmophiles, Institut Pasteur, 75724 Paris Cedex 15, France; 5 INRA, Unité MaIAGE, Université Paris-Saclay, 78352 Jouy-en-Josas CEDEX, France; 6 C2RT-Plateforme de cristallographie, Institut Pasteur, CNRS UMR 3528, 75724 Paris Cedex 15, France; 7 Unité de Biologie et Génétique de la paroi bactérienne, Institut Pasteur, INSERM équipe Avenir, 75724 Paris Cedex 15, France

## Abstract

Upon triggering by their inducer, signal transduction ATPases with numerous domains (STANDs), initially in monomeric resting forms, multimerize into large hubs that activate target macromolecules. This process requires conversion of the STAND conserved core (the NOD) from a closed form encasing an ADP molecule to an ATP-bound open form prone to multimerize. In the absence of inducer, autoinhibitory interactions maintain the NOD closed. In particular, in resting STAND proteins with an LRR- or WD40-type sensor domain, the latter establishes interactions with the NOD that are disrupted in the multimerization-competent forms. Here, we solved the first crystal structure of a STAND with a tetratricopeptide repeat sensor domain, PH0952 from *Pyrococcus horikoshii*, revealing analogous NOD-sensor contacts. We use this structural information to experimentally demonstrate that similar interactions also exist in a PH0952 homolog, the MalT STAND archetype, and actually contribute to the MalT autoinhibition *in vitro* and *in vivo*. We propose that STAND activation occurs by stepwise release of autoinhibitory contacts coupled to the unmasking of inducer-binding determinants. The MalT example suggests that STAND weak autoinhibitory interactions could assist the binding of inhibitory proteins by placing in register inhibitor recognition elements born by two domains.

## INTRODUCTION

Signal transduction ATPases with numerous domains (STAND) are a family of AAA+ related ATPases involved in a wide range of cellular activities (1,2). Upon activation by the cognate inducer molecule, these proteins build up multimeric hubs that trigger a signaling cascade. In the absence of inducer, STAND proteins are generally maintained in a monomeric autoinhibited resting form by numerous intramolecular interactions as well as interactions with inhibitory molecules. Eukaryotic STAND proteins comprise proapoptotic proteins like mammalian APAF1, *Drosophila* DARK and *Caenorhabditis elegans* CED-4, innate immunity receptors like the mammalian NOD1 and NOD2 proteins, as well as plant disease resistance R-proteins. Bacterial members of the STAND superfamily are mainly transcriptional activators such as the well-known *Escherichia coli* maltose system regulator MalT and serine-threonine kinases.

The hallmark of STAND ATPases is a conserved core called nucleotide-binding oligomerization domain (NOD), which is responsible for nucleotide binding and protein oligomerization. The NOD comprises the NBD-HD (nucleotide-binding domain-helical domain) module of AAA+ proteins (3) fused to a STAND-specific WHD (winged-helix domain) at the C-terminus. In most cases, the NOD is followed by an arm domain and a non-conserved sensor domain made of repeated motifs, which was found to contain the primary inducer-binding site in several instances (4–7). Finally, STAND ATPases generally contain at least one effector domain that is located at either protein end: this domain triggers downstream signaling upon protein activation.

The basal STAND switch, which relies on the particular architecture of the NOD, is conserved throughout the family. The NOD toggles between a closed form where an ADP molecule is clamped between the NBD-HD and the WHD, and an open form where the WHD is displaced and the nucleotide is solvent-exposed. NOD opening allows the replacement of ADP by ATP (8,9). The ATP-bound forms then undergo head-to-tail multimerization with the ATP sandwiched between adjacent protomers, which generates the active hub. In the last years, this scenario was vastly supported by structural, genetic and biochemical evidence from proteins from different STAND clades, including MalT, APAF1, mammalian NLR and plant R proteins.

How STAND proteins are kept in the inactive form by intramolecular interactions in the absence of inducer and how inducer-binding triggers their activation are two related issues that remain elusive. Based on recent studies, a scenario is emerging, in which inducer binding occurs in two steps: (i) a low-affinity binding step involving a subsite of the inducer-binding site; (ii) a rearrangement of domains that unveils a full, high-affinity binding site and which is coupled to the disruption of autoinhibitory interactions (6,8,10–12). Autoinhibitory contacts keeping NOD in the closed form involve primarily the arm, as observed in the crystal structures of resting APAF1, NLRC4 and NOD2, but also the WD40 or LRR sensors of these proteins, to a lesser extent (13,14). In the case of STAND with a TPR sensor, the key player of the autoinhibition is the arm domain, whose toggling between interactions that keep the NOD closed and interactions that help binding the inducer is the basis of the coupling between inducer-binding and NOD opening (8). Since in STAND with other types of sensor domains, sensor–NOD interactions seem to play a role in autoinhibition, we set out to determine whether such contacts also exist in STAND with a TPR sensor. This family presents several interesting features: its architecture is supposed to be that of the last common ancestor of STAND proteins (15), and it is widespread in all kingdoms of life.

Here, we report the crystal structure of *Pyrococcus horikoshii* PH0952, which reveals the existence of contacts between the NBD and the TPR sensor in the resting form. Using this structure as a guide and applying a combination of genetic, biochemical and structural bioinformatics approaches, we identify the NBD and sensor patches that are involved in the autoinhibition of MalT, a homolog of PH0952 and one of the best studied STAND proteins. These results suggest that NBD–sensor autoinhibitory contacts are a general feature of STAND proteins, which was unexpected considering the variety of sensor domain types exhibited by that superfamily.

## MATERIALS AND METHODS

### Strain and plasmids

*E. coli* strain pop7415 = MC4100 *ΔmalB107 trp::[Kan*^r^*-malEpΔ92-lac]*_op_*ΔmalT220 ΔcsgA::aadA7* (Spec^r^) *aes::Tn10* (Cam^r^) *ΔmalY*::*Zeo*^r^ F+ (16). pOM258 and pOM260 are derivatives of the single-copy R1 run-away plasmid pJM241 (17) that contain the *malT* gene under the control of the constitutive P_KAB-TTGG_ and P_KAB-TTCT_ promoters (18), respectively. pOM168 is a pKYB1 (New England Biolabs) derived expression plasmid encoding a fusion between PH0952 devoid of its DNA-binding domain and the Sce VMA1 intein. pOM206 is a pET24a(+) (Novagen) derived expression plasmid encoding a His-tagged version of *E. coli* MalT. See Supplementary Materials and Methods section for more details on the plasmids.

### Protein purification

A PH0952 variant devoid of its DNA-binding domain (PH0952ΔN) was purified using the IMPACT™ system (New England Biolabs). Plasmid pOM168 was introduced in Rosetta™ (DE3), and the resulting strain was grown in ZYP5052 (containing 0.01% glucose instead of 0.05%) (19) autoinduction medium at 20°C for 20 h. For the purification of selenomethionine-substituted PH0952ΔN, Rosetta™ (DE3) (pOM168) was inoculated at OD 4.5 in a modified PASM5052 medium ((19), see Supplementary Materials and Methods section) containing 1 mM isopropyl-β-D-thiogalactoside (IPTG) and grown at 20°C for 20 h. The fusion proteins were adsorbed on chitin beads in 50 mM Tris buffer, pH 8.1, containing 10% sucrose, 0.35 M KI and 0.4 mM ATP. Intein cleavage was carried out in the same buffer supplemented with 50 mM dithiothreitol (DTT) during 24–48 h at 4°C, and the recovered protein was further purified by size-exclusion chromatography on a Superdex 200 column (GE Healthcare) in 20 mM N-cyclohexyl-2-aminoethanesulfonic acid (CHES) buffer, pH 9.5, containing 0.3 M KCl, 2 mM Mg acetate, 0.4 mM ADP and 1 mM DTT. TCEP (0.5 mM) was added to the protein solution just before crystallization.

His_6_-tagged MalT and variants MalT^M96T^, MalT^H562Q^, MalT^R171E^, MalT^M96T,R171E^, MalT^M96C^, MalT^H562C^ or MalT^M96C,H562C^ were purified from pop8012 (a BL21 *ΔmalT* strain, (20)) harboring the relevant pOM206 (20) derivatives and grown in ZYP5052 at 20°C, by nickel-agarose chromatography followed by size-exclusion chromatography on a Superdex 200 column. The superdex mobile phase for MalT^M96C^, MalT^H562C^ and MalT^M96C,H562C^ was a Tris-HCl buffer (50 mM, pH 8.0) containing 10% sucrose, 0.3 M KCl, 10 mM Mg acetate, 0.1 mM ethylenediaminetetraacetic acid (EDTA) and 0.4 mM ATP, while for MalT^M96T^, MalT^H562Q^, MalT^R171E^ and MalT^M96T,R171E^, it was a Tris–HCl buffer (50 mM, pH 8.0) containing 10% sucrose, 0.033 M K_3_ citrate, 10 mM Mg acetate and 0.1 mM EDTA. MalT was purified in both conditions to serve as a control.

### PH0952 crystallization, data collection and structure determination

All crystallization trials were carried out using the sitting-drop vapor diffusion method and a Mosquito nanoliter-dispensing crystallization robot (TTP Labtech), by mixing 300 nl of protein solution and 300 nl of reservoir solution, equilibrated against 150 μl of reservoir solution in Grenier 96-well plates. Crystallization plates were stored at 18°C in a RockImager 1000^TM^ (Formulatrix, USA) automated imaging system to monitor crystal growth. The optimized conditions for crystal growth were 10% (v/v) 2-propanol, 0.1 M 4-(2-hydroxyethyl)-1-piperazine ethanesulfonic acid (HEPES), pH 7.7, for seleno-methionine (Se-Met)-labeled PH0952ΔN (10 mg/ml) and 10% (v/v) 2-propanol, 0.1 M imidazole, pH 8.0, for native PH0952ΔN (15 mg/ml). Crystals grew after 10 days to a size of ∼100 x 100 x 100 μm^3^. For cryo-crystallography, crystals were soaked in a cryo-protectant solution composed of crystallization solution mixed with 30% (v/v) ethylene glycol and then flash-frozen in liquid nitrogen for data collection at 100 K.

Highly redundant X-ray diffraction data were obtained from eight Se-Met PH0952 crystals on beamline Proxima 1 (Synchrotron Soleil, Saint-Aubin, France). The wavelength for data collection was 0.97918 Å, corresponding to the peak of the Se K edge, as measured by a fluorescence scan. Datasets from single crystals were indexed and integrated with XDS (21) and scaled with XSCALE (22) to generate a multicrystal dataset in space group P6_5_22. The selenium-substructure determination was performed with SHELXD through HKL2MAP (23). Initial SAD phases were calculated by Phaser (24) and then subjected to automatic density modification with solvent flattening and histogram matching as implemented in the CCP4 program Parrot (25). The obtained electron density map was interpretable, allowing us to recognize the topology of two NOD and two TPR domains in the asymmetric unit. Using HHpred (26), we retrieved from the PDB the structural models with the highest similarity to PH0952 NOD and TPR domains, respectively the NOD domain of APAF1 (PDB code: 1Z6T, 17% sequence identity, *E*-value 3.0 × 10^−16^) and the TPR domain of protein Pins (PDB code: 4A1S, 14% sequence identity, *E*-value 1.9 × 10^−18^). These models were rigid-body fitted into the experimental electron density using the program Coot (27). Notably, the position of PH0952 Met residues as predicted by sequence alignments with APAF1 NOD and Pins TPR domains perfectly matched positive peaks in a difference anomalous map calculated with diffraction data from Se-Met PH0952. The arm domain was manually built with Coot, and model improvement was performed by iterative rounds of manual model building and crystallographic refinement. High R factors in reciprocal space refinement cycles suggested that the choice of space group P6_5_22 was possibly incorrect. Indeed, data reduction in space group P6_5_ (Table 1) followed by data analysis with Phenix.Xtriage (28) suggested the presence of merohedral twinning (twin law h,-h-k,-l).

**Table 1.** Data collection and refinement statistics

	Se-Met PH0952	Native PH0952 (PDB code: 6MFV)
**Data collection**		
Space group	P6_5_	P6_5_
Cell dimensions		
*a, b, c* (Å)	98.43 98.43 589.42	96.13 96.13 584.3
*α, β, γ* (°)	90 90 120	90 90 120
Resolution range (Å)	49.21–3.70	48.69–3.40
*R*_merge_ (within I+/I-)	0.135 (2.133)*	0.083 (0.716)
*R*_merge_ (all I+ and I-)	0.155 (2.331)	0.093 (0.791)
*R*_pim_ (within I+/I-)	0.029 (0.442)	0.057 (0.490)
*R*_pim_ (all I+ and I-)	0.023 (0.340)	0.042 (0.355)
*I* / σ*I*	12.2 (1.8)	8.9 (1.4)
Completeness (%)	100 (100)	100 (100)
Multiplicity	47.0 (48.4)	5.8 (5.8)
**Refinement**		
Resolution (Å)		48.06–3.4 (3.521–3.4)
No. of reflections		41668 (4177)
*R*_work_/*R*_free_		0.2244/0.2716
Protein residues		2564
Ligand molecules		4
No. of atoms		
Protein		21 276
ADP		108
Wilson *B*-factor (Å^2^)		104.65
*B*-factors (Å^2^)		
Protein		150.43
ADP		102.78
R.M.S. deviations		
Bond lengths (Å)		0.004
Bond angles (°)		0.97

*Values in parentheses are for highest resolution shell.

PH0952 crystals were isomorphous to Se-Met PH0952 crystals (Table 1). X-ray diffraction data from a single native PH0952 crystal were collected at the synchrotron beamline Proxima 2 (Synchrotron Soleil, Saint-Aubin, France) using wavelength 0.9801 Å. The diffraction data were processed using XDS (21) and scaled with Aimless (29). The initial model of Se-Met PH0952 built as described above was used to phase the diffraction data from native PH0952. Model improvement was performed by iterative cycles of manual model building with Coot (27) and reciprocal space refinement with twin law h,-h-k,-l using Phenix.Refine (28). ADP molecules were manually placed in mFo–DFc sigma-A-weighted electron density maps. Refinement of the final PH0952 model (Table 1) back in space group P6_5_22 resulted in R_work_/R_free_ values of 0.42/0.38, supporting the choice of space group P6_5_ for data. Additionally, model refinement in space group P6_5_ with no twin law gave *R*_work_/*R*_free_ values of 0.35/0.31. The final model was validated through the Molprobity server (http://molprobity.biochem.duke.edu) (30). It contains more than 96% of residues within favored regions of Ramachandran plot and <0.05% of Ramachandran outliers. Figures were generated and rendered with Pymol 1.5.0.2. (Schrödinger, LLC). Atomic coordinates and structure factors have been deposited in the Protein Data Bank under the accession code 6MFV.

### Phylogenetic and clustering analyses

Protein sequences representing previously defined families and clades of STAND ATPases were collected by BLASTP searches against the nr50 UniProt database. PH0952 homologs were retrieved from the non-redundant protein database at NCBI. Sequences from each clade were aligned separately and the resultant alignments were merged using MAFFT (31). The uninformative positions were removed using the strict function of the trimAL program (32). Phylogenetic trees were constructed using PhyML (33) with an automatic selection of the best-fit substitution model for a given alignment (LG +G+F). A Bayesian-like transformation of aLRT (aBayes), as implemented in PhyML, was used to estimate branch support. The best-fit substitution model for the PH0952 orthologs from Thermococcales was found to be LG +G+I+F.

The sequences were clustered by similarity (BLOSUM62 matrix, *E* = 10^−3^) using the CLANS program that generates a network representation of pairwise sequence similarities between proteins using a version of the Fruchterman-Reingold graph layout algorithm (34).

### Homology modeling of MalT

In a first step, the structure of MalT devoid of its C-terminal DNA-binding domain was homology-modeled, using the model-building software Modeller (mod9v18) (35). The crystal structure of PH0952 solved in this work served as a 3D template to model the NOD–arm segment of MalT (residues 1–442) (16% identity, *E*-value 6.8 × 10^−32^ as determined by HHpred) and the crystal structure of the TPR sensor domain of MalT (PDB code: 1HZ4, (36)) served as a 3D template for the region encompassing residues 443–803 of the model. The two sequences were merged in the alignment file to generate a MalT model from residue 1 to 803.

In the second step of modeling, results of the cysteine pair cross-linking assay reported here and in previous work (8) were used to refine the model. Three successive rounds of modeling (100 models each) were applied: first, models of MalT^Q70C,M96C,E395C,H562C^ with two disulfide bridges were generated; second, the best model (according to the DOPE and Modeller score functions (35)) of round 1 was used as a template along with the PH0952ΔN and MalT sensor domain crystal structures to model the wild-type protein, with a constraint on the Cα–Cα distances of Q70-E395 and M96-H562; third a final modeling was performed without distance constraints, starting with the best model obtained in round 2. The final model was the one with the lowest score function values and best stereochemistry, checked by Molprobity (http://molprobity.biochem.duke.edu/) (30).

### Screening for gain-of-function *malT* mutations altering the NBD or the sensor domain

Mutations *M96T* and *H102Y* were isolated as described by Liu *et al.* (16), i.e. by random PCR mutagenesis targeted at the *malT* region encoding residues 7–207 encompassing most of the NBD. Mutations *H562Q, H562R* and *Q565R* were isolated by using strain pop7192 as described in Richet *et al.* (37), and applying the same mutagenesis technique to the *malT* region encoding the 431–826 polypeptide, which encompasses the sensor domain. As described, the mutagenized DNA fragments were reintroduced in an otherwise wt *malT* gene borne by a low-copy plasmid, and the obtained plasmid bank was screened for constitutive activation of a MalT-dependent promoter in the absence of external inducer.

### β-Galactosidase assays

*E. coli* strain pop7415 harboring pJM241 or pOM258 derivatives was grown in minimal M9 medium supplemented with 0.4% glycerol, 0.01% tryptophan, 1 μg/ml thiamine and 30 μg/ml ticarcillin, as described by Liu *et al.* (16). β-Galactosidase activity was assayed in duplicate on each culture as described, and the obtained value was corrected for the background (9 Miller units) as measured with pop7415 (pJM241). The fold enhancement in the β-galactosidase activity was determined for each variant as the ratio of the variant activity to the activity of wild-type MalT (∼26 Miller units), as assayed on the same day. The means of the fold enhancement values ± SD were calculated from three independent series of cultures.

### Western blots

*E. coli* strain pop7415 harboring plasmids pJM241 or pOM260 derivatives was grown as described above, and total cell extracts were analyzed on a 10% SDS-polyacrylamide gel. Proteins were transferred on a Hybond^TM^ ECL membrane, and probed with rabbit polyclonal anti-MalT followed by a horseradish peroxidase-conjugated secondary antibody-based ECL Plus detection on a Typhoon imager (GE healthcare). Relative amounts of the variant MalT protein levels with respect to that of wild-type MalT were determined as the ratio of the MalT band volumes after correction for the background value measured with pop7415 (pJM241). Immunoblot analyses were performed under linear dose–response conditions. Mean values ± SD of the relative amounts of the MalT variant protein levels were calculated from three independent series of cultures. Under the growth conditions used, pop7415 (pOM260) produces ∼10-fold more MalT than pop7415 (pOM258).

### Analytical size-exclusion chromatography

Proteins were incubated for 10 min at room temperature in the presence of the indicated effectors and injected on a Superdex 200 PC3.2/30 column mounted on an Ettan LC system (GE Healthcare) run at room temperature and at a 40 μl/min flow rate.

### Cysteine pair cross-linking assay

Proteins MalT, MalT^M96C^, MalT^H562C^ and MalT^M96C,H562C^ were preincubated at 0.44 or 4.4 μM for 10 min at 23°C in 18 μl of Tris–HCl (55 mM, pH 8.0) containing 11% sucrose, 0.033 M K_3_ citrate, 0.015 M KCl, 9.5 mM Mg acetate, 0.42 mM ATP and 0.01 mM EDTA. The buffer also contained maltotriose, glucose or maltose when required. After addition of 2 μl of DTT, orthophenanthroline-copper (OP-Cu), cystamine/DTT or H_2_O, the reaction was allowed to proceed at the same temperature for 10 min. It was stopped by adding sample buffer containing N-ethylmaleimide and EDTA, both at 10 mM final concentration. The extent of cysteine pair cross-linking was evaluated by non-reducing sodium dodecyl sulphate-polyacrylamide gel electrophoresis (SDS-PAGE) on 10% gels.

### Limited proteolysis

Proteins (4 μg) were preincubated for 10 min at 25°C in a buffer containing 50 mM HEPES (pH 8.0), 11 mM Tris–HCl (pH 8.0), 2% sucrose, 7 mM K_3_ citrate, 0.3 M KCl, 11 mM Mg acetate, 0.44 mM ATP or ADP, 1 mM DTT, 0.02 mM EDTA and maltotriose at 22 mM when required. Proteinase K was added, and the reaction was allowed to proceed for 30 min at 25°C. The reaction was stopped by precipitating the samples with 3 volumes of trichloroacetic acid 22%. The precipitates were recovered by centrifugation, washed with cold acetone, dried, redissolved in sample buffer containing 70 mM DTT and analyzed by SDS-PAGE on 10% gels.

## RESULTS

### Structure of the resting form of the *P. horikoshii* PH0952 protein, a MalT homolog

**Figure 1.**
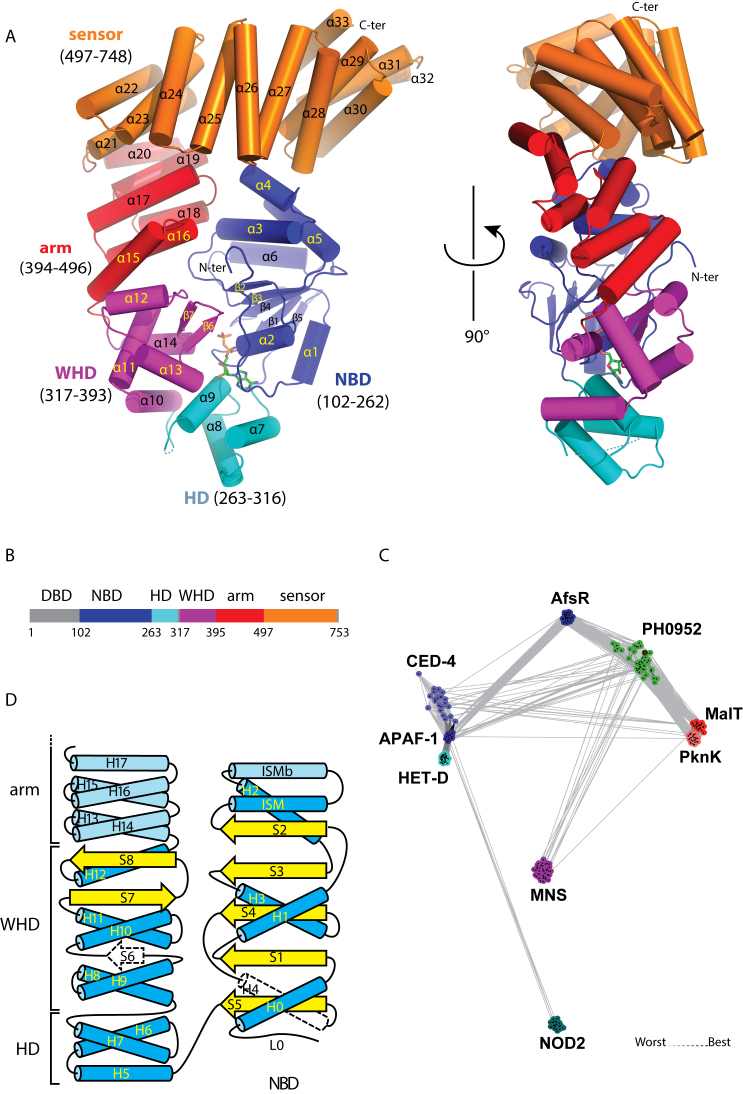
The *Pyrococcus horikoshii* PH0952 protein. (**A**) Overall fold of PH0952ΔN. The protein is depicted in cartoon representation with helices shown as cylinders (PDB code: 6MFV). (**B**) Primary structure of PH0952ΔN with the same color code as in (A). Regions not present (DBD) or not resolved in the crystal structure are represented in gray. (**C**) Sequence similarity networks of STAND ATPases. Protein sequences were clustered by the pairwise sequence similarity (*E* = 10^−3^) using CLANS. Different clades of STAND ATPases are shown as clouds of differentially colored circles identified by the name of a prominent clade member. The PH0952 protein is indicated by a black dot. (**D**) Secondary structure of PH0952 and its relationship with the general STAND fold (3). PH0952 α-helices (H) are in blue and β-strands (S) in yellow. Secondary structure elements present in the STAND fold but not in the PH0952 protein are represented in white with a dashed outline. Secondary structure elements present in PH0952 but not in all STAND proteins are represented in a paler shade. The numbering of the secondary structure elements refers to their sequence in the general STAND fold when possible.

To identify interactions between the NOD and the sensor domains of MalT possibly involved in the autoinhibition process, we first tried to solve the X-ray structure of MalT and close homologs from proteobacteria. As extensive attempts to crystallize these proteins failed, we screened different MalT homologs from archaea for crystallization. We obtained diffraction quality crystals of protein PH0952 from the euryarchaeon *P. horikoshii* lacking its effector domain (PH0952ΔN, Figure 1A). PH0952 is a predicted transcription factor with a TPR sensor domain and a putative N-terminal ArsR-like DNA-binding effector domain (Figure 1B) (38). Clustering of STAND proteins representing different clades and pairwise comparison (Figure 1C) show that PH0952 homologs are positioned between the APAF1 and MalT clades, and are only distantly related to other STAND clades. Consistently, in a phylogenetic analysis of the STAND superfamily (Supplementary Figure S1), the PH0952 clade formed a sister group to the APAF1 clade, with the MalT clade being at the base of the PH0952–APAF1-clade assemblage. Altogether, these analyses suggest that PH0952-like proteins represent a ‘missing link’ between the MalT and APAF1 clades.

Native crystals of PH0952 lacking its N-terminal effector domain (Table 1) belonged to space group P6_5_ and diffracted X-rays to 3.4 Å. The asymmetric unit contained four protein molecules with RMSD values lower than 0.38 Å among 641 αC atoms. Each PH0952ΔN molecule exhibits a curled up conformation in the shape of the letter G, with 90 × 60 × 40 Å^3^ dimensions (Figure 1A). In the putative nucleotide-binding site, a ligand was clearly visible in the experimental electron density map (Supplementary Figure S2) and was interpreted as an ADP molecule (present in the buffer). We cannot exclude that PH0952 binds GDP; however, most STAND proteins tested thus far preferentially bind (deoxy)adenine nucleotides (2,39). The domain organization of the protein is that of a bona fide STAND protein in the monomeric resting state (Figure 1A and B), consistent with the presence of a diphosphate nucleotide. It comprises a NOD module with three domains: the three-layered α/β nucleotide-binding domain (NBD, amino acids 102–262), the helical domain (HD, amino -acids 263–316) and the winged-helix domain (WHD, amino acids 317–393). The NOD is followed by an arm domain (also named HD2, amino acids 394–496) and a sensor domain (amino -acids 497–748) composed of tetratricopeptide repeats. Compared to the other STAND proteins for which a crystal structure comprising the sensor domain is known, i.e. APAF1 (PDB code: 3SFZ, (14)), NOD2 (PDB code: 5IRM, (39)) and NLRC4 (PDB code: 4KXF, (13)), the PH0952 NOD module and arm domain are structurally most closely related to those of APAF1 (Supplementary Figure S3), consistent with the phylogenetic and clustering analyses (Figure 1C and Supplementary Figure S1).

As in all STAND proteins, the NBD of PH0952 is built on a AAA+ scaffold composed of five parallel β-strands (β1–5, Figure 1A and Supplementary Figure S4, strands S1–5, Figure 1D) alternating with α-helices. These secondary structure elements form a core parallel β-sheet sandwiched between helix H1 on one side and all the other helices on the other side (Figure 1D). Note that throughout the text, secondary structure elements will be referred either with respect to the general STAND topology as described in Figure 1D (with Hn standing for helix n and Sn for β-strand n) or according to their succession in the PH0952 structure (Figure 1A) (with αn standing for helix n and βn for β-strand n). The PH0952 AAA+ scaffold is modified by the following features: (i) two α helices are inserted between β-strand S2 (β2) and helix H2 (α5). The first one, α3, parallel to helix α5 (H2), corresponds to the conserved ISM helix that features the STAND as well as the AAA+ initiator clade NBDs (3); the second one, α4 (ISMb) is antiparallel to ISM and helix α5 (H2); it is also present in NOD2 but is replaced by a loop in APAF1 and NLRC4. (ii) Helix H4 is replaced by an unstructured region in PH0952.

The PH0952 HD is composed of the three helices (H5–H7) that represent the basic fold of the HD (the APAF1 HD derives from it by the addition of a helix after H7, while NOD2/NLRC4 have a small helix inserted between H6 and H7). The HD is connected by a 6 amino acid unresolved stretch to the WHD, which is a typical winged-helix domain except that the S6 β-strand connecting H9 and H10 (α11 and α12, Figure 1A and D) is replaced by a loop. The PH0952 WHD superimposed well with those of APAF1, NOD2 and NLRC4. One difference between the WHDs of APAF1/PH0952 and NOD2/NLRC4 is the longer loop at the end of the S7-S8 ‘wing’ (or β-hairpin) of the latter ones.

The PH0952 arm domain is composed of three helical hairpins arranged in a right-handed solenoid, reminiscent of tetratricopeptide repeats (Figure 1A) (40). Consistently, the closest homologs retrieved by a Dali (41) search using the PH0952 arm as a query are TPR and TPR-like domains (Supplementary Figure S5). The APAF1, NLRC4 and NOD2 arm domains may appear as variations of this TPR-like module by motif insertion and/or deletion (Supplementary Figure S6), with the APAF1 arm being most similar to the PH0952 arm. It is therefore tempting to speculate that the arm evolved from a TPR domain, on which different structures have been grafted as the proteins diverged and specialized through the acquisition of sensor domains of different kinds. The fact that the sensor domain of the ancestor of STAND proteins was composed of TPR repeats (15) raises the interesting possibility that the arm is a remnant of the TPR repeats of this ancestral protein.

Finally, the sensor domain of PH0952 is a typical TPR module with six tetratricopeptide repeats (α21–α32, Figure 1A) and a capping helix (α33) characteristic of TPR domains (42).

### ADP establishes interactions with all the subdomains of the NOD

**Figure 2.**
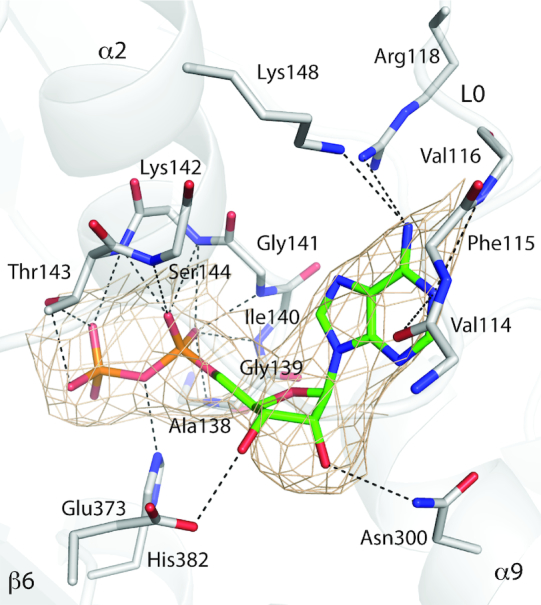
The ADP-binding site of PH0952. The protein is depicted as gray ribbons. The ADP molecule and most protein residues (main chain atoms or side chains) interacting with it are shown in sticks with atoms colored as follows: C in green for ADP and gray for the protein, N in blue, O in red and P in orange. The 2mF_o_–DF_c_ electron density is contoured to 1.3 σ and represented as a beige mesh. Dashed lines represent hydrogen bonds (PDB code: 6MFV).

The environment of the ADP molecule in PH0952 is similar to that in APAF1 (43). Its adenosine moiety is sandwiched between two sets of secondary structure elements: the L0 loop upstream of helix α1 (H0 in the STAND fold) and helix α2 (H1) of the NBD on one side, helix α7 (H5) and α9 (H7) of the HD on the other side (Figure 2). Thus, the adenosine ring establishes hydrogen bonds and hydrophobic interactions with PH0952 residues 114–118, 141, 144, 148, 272, 296 (belonging to the conserved GxP motif (1)) and 300. The ribose moiety interacts through hydrogen bonds with residues 300 and 373 in the HD and the WHD, respectively. As expected, the α and β phosphates establish hydrogen bonds with residues 139–144 in the P-loop and residue H382 in the WHD. In NLRC4 and NOD2, a region hereinafter called R0, comprised of several secondary structure elements, replaces loop L0 and plays the same role, and the ribose moiety does not appear to form hydrogen bonds with the protein directly (13,39).

### Interdomain interactions in the crystal structure of PH0952ΔN

Consistently with phylogenetic analyses, interactions between consecutive domains of the NOD–arm module are extremely conserved between PH0952 and APAF1, but less so between PH0952 and NOD2 or NLRC4 (Supplementary Figure S3). As in APAF1, the PH0952 NBD-HD interface involves packing of loop L0 against HD helix H5, and interaction between the P-loop of the NBD and the conserved GxP motif of HD helix H7 (Supplementary Figure S3). In NOD2/NLRC4, the role of loop L0 is played by the R0 substructure (Supplementary Figure S3). At the HD–WHD interface of APAF1 and PH0952, HD helix H7 rests on WHD helix H8 (PH0952 α10) and the C-terminal tip of WHD helix H11. In the NLRC4/NOD2 structures, the interface is less extensive (39), mainly due to a different relative orientation of these two domains: only helix H8 of the WHD interacts with the HD. Finally, the PH0952 WHD-arm interface is characterized by the docking of H10 (α12) and the H9-H10 (α11-α12) loop of the WHD against arm helices H13 (α15) and H14 (α16), respectively. In APAF1, the same elements (with β-strand S6 replacing the H9-H10 loop) interact in a slightly different way (Supplementary Figure S3).

In the PH0952 arm, the packing of the TPR-like helical hairpins differs from that of classical TPR repeats, and this affects the arm-sensor interface. In classical TPR repeats, the helical hairpins are parallelly stacked into a slab with one face built by the first helices (helices A) and the other built by the second helices (helices B). This slab is generally curved toward the A side (40,44) because BAB angles (angles between the directions of the turns that bracket an A helix, projected down the helix axis, Supplementary Figure S7) are larger than ABA angles. This rule applies to most of the PH0952 arm, except for the BAB angle at helix α17 that is smaller than the ABA angles (Supplementary Figure S7), resulting in an overall slight curvature toward the B face. Hence, a change in the slab curvature occurs at the junction between the arm and the sensor domain of PH0952, since the latter has the normal TPR curvature. As a result, helices α17 and α19 in the arm and helices α21 and α23 in the sensor pack together burying hydrophobic groups, reminiscent of the arm–sensor interface in the NLRC4 protein ((13) and Supplementary Figure S7).

**Figure 3.**
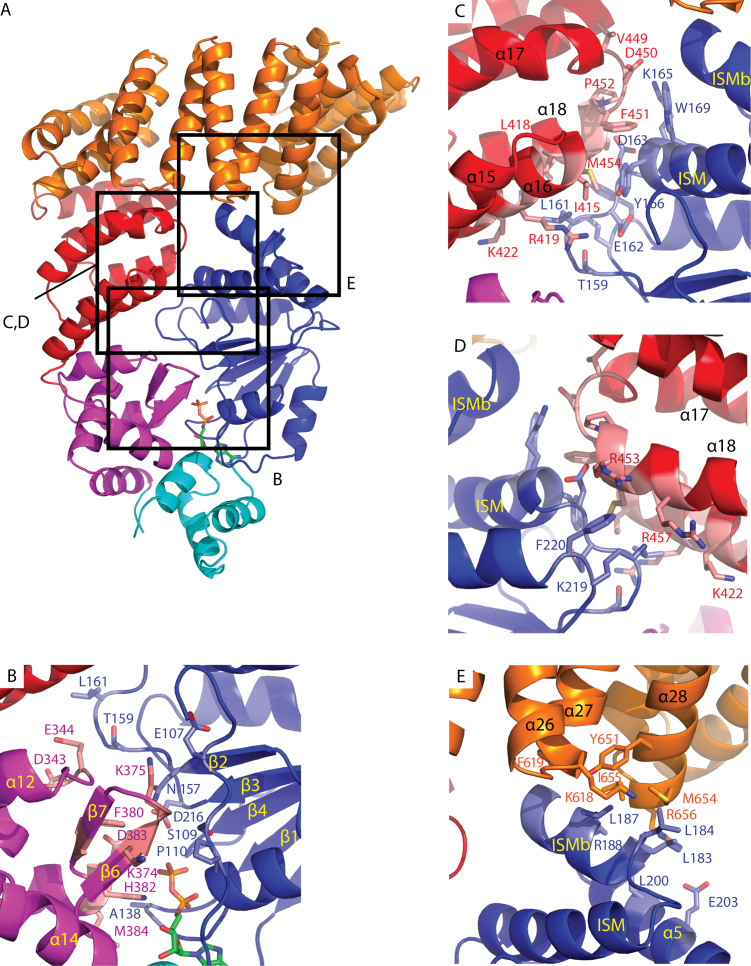
NBD interactions with non-adjacent domains in the PH0952 crystal structure. The protein is depicted in cartoon representation (PDB code: 6MFV). Highlighted residues are shown in sticks in the zoomed panels. (**A**) Overall view of PH0952ΔN. Squares highlight regions enlarged in the other panels. (**B**) NBD–WHD interface, (**C**) NBD–arm interface, (**D**) NBD–arm interface, rear view and (**E**) NBD–sensor.

Interfaces between the NBD and non-adjacent subdomains, which seem to play a role in STAND autoinhibition, are also well conserved between PH0952 and APAF1 (Figure 3A). Noteworthy, they are conserved between the four molecules present in the PH0952 crystallographic unit, suggesting that they do not result from crystal packing constraints. The interface between the WHD and the NBD of PH0952 (Figure 3B) involves a network of interactions connecting (i) the tip of the WHD β6–β7 (S7–S8) β-hairpin (residues 374–376) with the L0 loop (residues 107–110) and residue 157 in the C-terminus of strand β2 (S2) in the NBD; (ii) the N-terminus of helix α14 (H12) in the WHD with the P-loop and residue 216 in the C-terminus of strand β3 (S3) in the NBD; (iii) possibly the α11–α12 (H9–H10) turn in the WHD (residues 343–344) with loop 158–161 at the C-terminus of β2 (S2) in the NBD. Altogether, the interface area between the WHD and the NBD of PH0952 is close to 370 Å^2^. The architecture of this interface is relatively conserved in all STANDs crystallized in the resting form (13,39,43), with the role of the L0 loop being played by part of the R0 region in NOD2 and NLRC4. The arm–NBD interface in PH0952 (Figure 3C and D) relies on the packing of the first two helical hairpins of the arm against the NBD so that helix α18 (H16) stacks on the tip of helix ISM, with a similar direction but opposite polarity, similar to the situation in APAF1 (Supplementary Figure S3). The buried surface area between the arm and the NBD of PH0952 is 490 Å^2^, consistent with the key role of the arm in the STAND autoinhibition process. Compared to PH0952/APAF1, the position of the arm is radically different in NLRC4/NOD2, where the axis of the H13 arm helix is roughly perpendicular to its counterpart in PH0952/APAF1 and where the arm consequently interacts with a different face of the NBD (Supplementary Figure S3) (13,39). Finally, the PH0952 TPR sensor caps the tip of the NBD that is formed by helices ISM (α3), ISMb (α4) and H2 (α5) (Figure 3E), burying a surface of 360 Å, similar to the NBD–WHD and NBD–arm interfaces. More specifically, the NBD ISMb is wedged between the two helices of the fourth repeat of the TPR module (α27 and α28). Thus, the NBD is clamped between the arm and the sensor domain, suggesting that in the crystal, PH0952 is autoinhibited by NBD–arm and NBD–sensor interactions. Note that NBD–sensor interactions in resting APAF1, which are less extended than in PH0952, also involve the ISM helix (14,45), despite the absence of homology between the sensor domains in both proteins.

### Modeling of MalT after PH0952

Using homology modeling, we built a model of MalT containing the NOD, arm and sensor domains (residues 1–803). The templates used for the modeling were the NOD–arm part of the crystal structure of PH0952 (corresponding to residues 1–442 in the MalT model) and residues 6–366 of the crystal structure of the sensor domain of MalT (PDB code: 1HZ4, (36) corresponding to residues 443–803 in the MalT model). Models with the best score and stereochemistry (checked by Molprobity (30)) consistently displayed contacts between the NBD and the sensor domain involving the ISM helix in the NBD and the turn between repeats 3 and 4 in the TPR module (α6 and α7 of the MalT sensor). The slight shift between the NBD–sensor interface regions predicted for MalT and observed in PH0952 is explained by the more pronounced curvature of the TPR-like sensor in MalT (36).

### Gain-of-function mutations can be isolated in the NBD and in the sensor domain of MalT

If there is a contact between the sensor and the NBD in MalT and if this interaction is involved in the autoinhibition of the protein, then mutations altering this contact should result in a (partially) constitutive phenotype. To test this prediction, we first screened for gain-of-function mutations affecting the NBD and the sensor as described in Liu *et al.* (16) and Richet *et al.* (37), respectively. Besides mutations affecting other steps of the MalT signaling pathway (e.g. the NBD–WHD interaction (16)), we found five point mutations that alter the NBD–sensor interface predicted by our homology model: they resulted in amino acid substitutions M96T and H102Y, which affect the ISMb, and H562Q, H562R and Q565R which affect the α6-α7 turn of the MalT sensor. These mutations define two surface patches (referred to the M96 patch and the H562 patch), which display complementary charges (Supplementary Figure S8). Importantly, two of these substitutions, H562R and Q565R, decrease the negative charge of the sensor patch, consistent with the idea that they alter the hypothetical NBD–sensor interaction.

**Figure 4.**
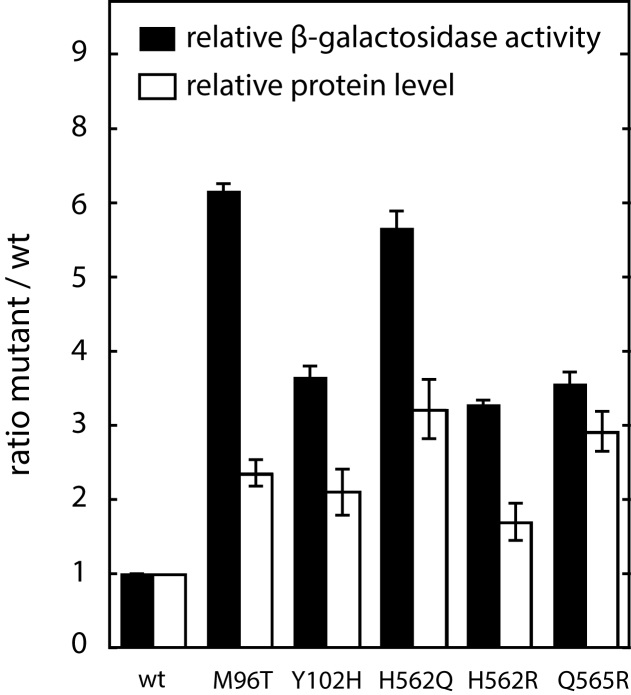
*In vivo* activities of the gain-of-function MalT variants. The activities of the gain-of-function MalT variants were determined by measuring the β-galactosidase activity of strain pop7415 harboring pJM241, pOM258 or derivatives thereof and grown in minimal medium supplemented with glycerol. The ratios of the variant activity levels to that of the wild-type activity level were calculated as described. The relative levels of the MalT variant proteins were determined by immunoblot analyses (see ‘Materials and methods’ section) in strain pop7415 harboring pJM241, pOM260 or derivatives thereof and grown under the same conditions.

After reintroducing these mutations in a clean genetic background, we assessed their effect on basal MalT activity in the absence of MalT inhibitory proteins (2) by using a chromosomal *lacZ* reporter gene placed under the control of a MalT-dependent promoter. All the above mutations conferred a higher expression (Figure 4), although the increase was generally smaller than that observed for other autoinhibition mutations, like those affecting the NBD–WHD interaction (16). Note that the gain-of-function substitutions reported here, like those affecting the NBD–WHD interface, increased the total level of MalT, suggesting that the active form of the protein is more resistant to proteolysis than the resting form. In conclusion, we found gain-of-function mutations affecting NBD and sensor residues that lay spatially close in our structural model; therefore, these residues are good candidates to participate in an NBD–sensor interaction in MalT.

### MalT residues M96 and H562 are in physical proximity when the protein is in the resting form

**Figure 5.**
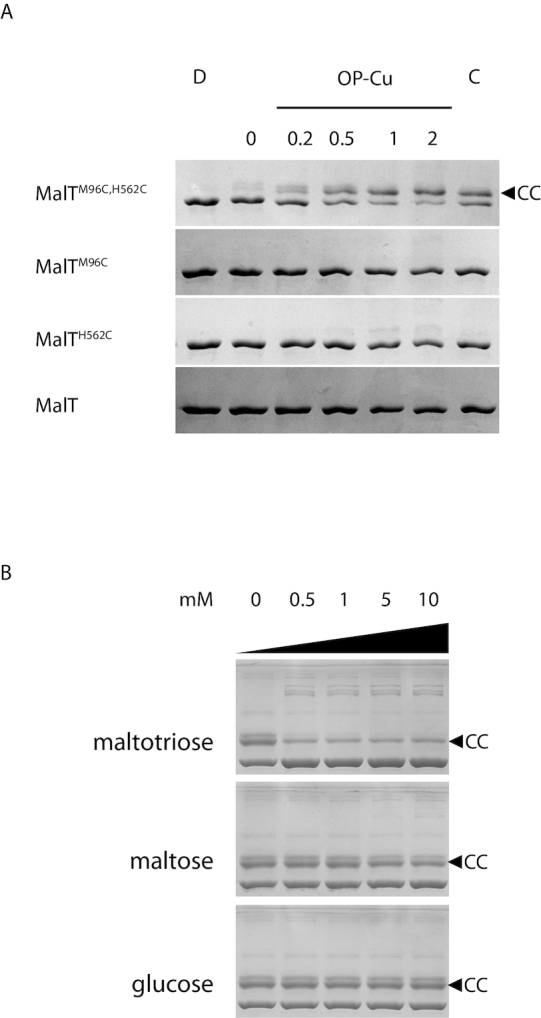
An inducer-sensitive disulfide-bond is formed specifically when both M96 and H562 of MalT are replaced by cysteines. (**A**) Proteins (0.4 μM) were incubated with 1 mM DTT (D), no reagent (0), the indicated concentration of orthophenanthroline-copper (OP-Cu, in μM) or with 0.5 mM:0.05 mM cystamine/DTT (C) and analyzed by SDS-PAGE. (**B**) MalT^M96C,H562C^ (4 μM) was preincubated with the indicated concentration of carbohydrate and probed with 2 μM OP-Cu. CC indicates the band corresponding to MalT^M96C,H562C^ protein in its disulfide-bonded form. Note that at the low protein concentrations used, the protein is known to remain in the resting form in the presence of ATP alone and requires both maltotriose and ATP for multimerization (48).

In a second step, we examined whether the two surface patches identified in the genetic screens are physically close in the MalT resting form, by using a cysteine-based cross-linking approach (46). We mutagenized the *malT* gene to create alleles coding for MalT proteins bearing either one cysteine at position 96 or 562, or cysteines at both positions, and we purified the three variants and MalT in the same conditions. The mutations did not interfere with MalT activation in reducing conditions. Indeed, the four proteins were monomeric in the absence of the inducer, maltotriose (47), and responded to its presence by multimerizing to the same extent (Supplementary Figure S9). To determine whether cysteines C96 and C562 introduced in MalT^M96C,H562C^ are able to form a disulfide bond, we used SDS-PAGE migration of the purified proteins as a read-out (Figure 5). We incubated MalT and its three variants in conditions in which the protein remains in the resting form, with a reducing agent (DTT), with an oxidation catalyst (orthophenanthroline-copper (OP-Cu)), with an oxidant (cystamine) or without any reagent. In the presence of DTT, the four proteins migrated at the same level (Supplementary Figure S10). In the absence of any reagent, a faint band migrating more slowly was detected exclusively for the MalT^M96C,H562C^ protein (Figure 5A), probably due to oxidation by the oxygen present in the incubation buffer, as already observed for other cysteine cross-linking experiments on MalT (8). This shifted band became more prominent with increasing concentrations of OP-Cu, until it comprised more than half of the total protein. Its intensity was also increased by the addition of the unrelated oxidant cystamine, indicating that oxidation per se was the reason for its differential migration. Importantly, the migration patterns of the single mutants and the wild-type were virtually unaffected by oxidation. A very faint band was visible only at the highest OP-Cu concentrations in the MalT^H562C^ lane, which could be explained by disulfide bridges formed between C562 and cysteines at positions 89 and 93, close to the M96 residue. In conclusion, oxidation causes a shifted band to appear when two cysteines are present at positions 96 and 562, but not when only one or none of them is present. Formation of a disulfide bond between C96 and C562 suggests that M96 and H562 lie physically close to each other in the resting form of the wild-type MalT protein.

If an interaction between the M96 and H562 patches is involved in MalT autoinhibition, this contact should be disrupted in the active form of the protein. To check whether the formation of the C96-C562 disulfide bond of the MalT^M96C,H562C^ variant is specific of the resting form, we examined whether it can form if the protein is activated by adding its inducer. The formation of the disulfide bond catalyzed by OP-Cu was analyzed in the presence of increasing maltotriose concentrations. As predicted, maltotriose reduced the formation of the 96–562 disulfide bond, even at the lowest concentration tested (Figure 5B). No such effect was observed at 20 times higher concentrations of maltose or glucose, demonstrating that this effect is specific for the cognate inducer of the protein. Altogether, these results show that residues 96 of the NBD and 562 of the sensor lie physically close and that this proximity is specific of the resting form. The faint bands of higher apparent molecular weight that appear in the presence of inducer are most likely explained by MalT multimerization followed by disulfide cross-linking of the protomers.

### The M96T single substitution affects MalT autoinhibition

Finally, to obtain direct evidence that the M96 patch is involved in the autoinhibition of MalT, we set out to determine whether the gain-of-function M96T substitution destabilizes the resting form *in vitro* (the gain-of-function phenotype observed *in vivo* could be due to the higher protein level observed). We therefore purified the MalT^M96T^ protein along with the wild-type protein and compared their ability to multimerize in the absence of inducer by analytical size-exclusion chromatography.

MalT multimerization is a highly dynamic process: MalT protomers associate head-to-tail into heterodisperse multimers that are in rapid equilibrium on the timescale of the chromatography and whose average size is a function of protein concentration (48) (see also Supplementary Figure S9). As a result, the protein dilution that occurs during size-exclusion chromatography gives rise to a peak with a steep front and a trailing end, with the elution volume of the peak reflecting a weighted average of the Stokes radii of the multimers in equilibrium.

**Figure 6.**
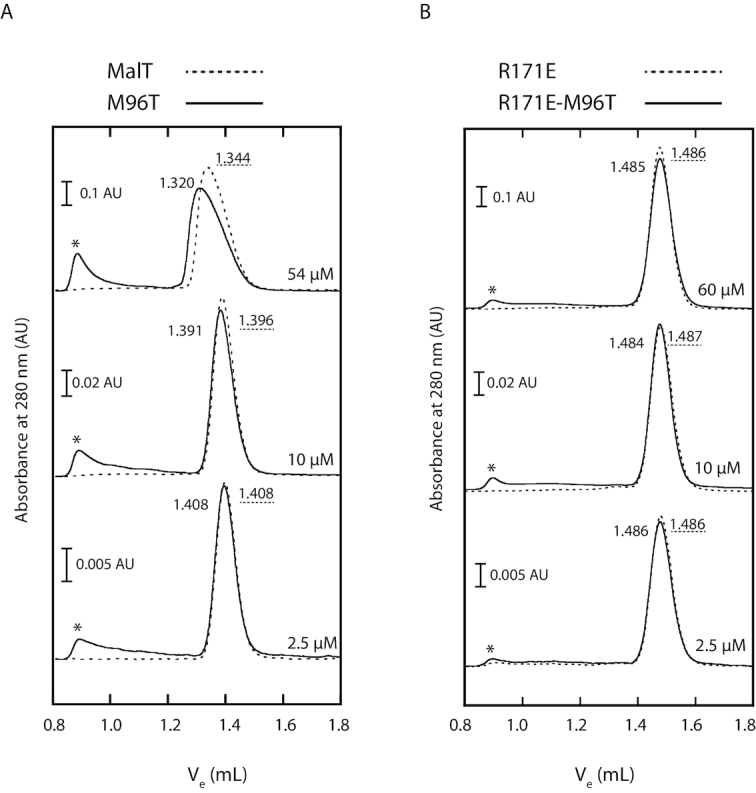
The M96T substitution increases native MalT-ADP multimerization in the absence of maltotriose. (**A**) After a 10-min preincubation in a Tris–HCl buffer (50 mM, pH 8.0) containing 10% sucrose, 0.033 M K_3_ citrate, 10 mM Mg acetate, 0.1 mM EDTA, 1 mM dithiothreitol and 0.1 mM ADP, MalT or MalT^M96T^ were injected at the indicated concentration on a Superdex 200 column equilibrated with the same buffer. The MalT^M96T^ concentration was adjusted to account for the presence of aggregates (eluting as peak in the void volume, marked by a star), probably due to partial opening of the protein and release of the bound ADP during the purification in the absence of nucleotide. Note the different scales. The 54 and 2.5 μM experiments were repeated three times by alternating the two proteins on the same column. For these experiments, the mean elution volume (ml) is indicated (with dotted underline for MalT). Variations in the elution volumes between the three repeats did not exceed 0.002 ml. (**B**) MalT^R171E^ (elution volumes with dotted underline) or MalT^M96T,R171E^ was preincubated and injected as above on a Superdex 200 column with the same characteristics but with a shifted calibration curve (see Supplementary Figure S12) due to a longer time of use. On that column, the elution volume for a wt MalT monomer (in the same conditions, protein concentration 10 μM) was 1.484 ml. MalT^M96T,R171E^ aggregates are also marked by a star. The 60 μM experiments were repeated twice and the variations did not exceed 0.003 ml.

To increase the chances of observing an effect of the M96T substitution, we chose conditions resulting in a low degree of multimerization: the proteins were assayed in the absence of maltotriose, and in the presence of ADP instead of ATP. The inducer was omitted because it favors a conformation in which the M96 and H562 patches are apart. ADP supports multimerization, albeit at a higher protein concentration compared to ATP (48). To eliminate ATP traces, the proteins were purified in the absence of nucleotide, which resulted in partial (20%) aggregation of MalT^M96T^, as already observed with MalT variants exhibiting a constitutive activity (16,20,49). Protein concentration was corrected to account for this phenomenon. As expected, in these conditions, MalT behaved as a monomer at low concentration and moderately multimerized at higher concentrations, as recognized by the wider, asymmetrical and shifted peak typical of MalT oligomers (48) (Figure 6A). While MalT^M96T^ behaved like MalT at low concentration, it clearly associated into larger oligomers at high concentration (Figure 6A, top curves).

The change in the chromatography profile observed for the M96T variant did not result from an effect of the M96T substitution on protein folding, protein tertiary structure or on the maltotriose-induced conformational changes, as shown by limited proteolysis of MalT and MalT^M96T^ (Supplementary Figure S11A). Indeed, wild-type MalT in its resting form is characterized by proteolysis hypersensitivity of the arm–sensor hinge, which generates proteolytic fragments of apparent molecular weights 50 and 45–48 kDa (5,8). By contrast, when MalT is in the activated, multimerization-competent form, proteolysis occurs at the HD–WHD hinge, producing two fragments (66 and 25 kDa (the latter barely visible)). As expected, similar profiles were observed for the M96T variant (Supplementary Figure S11A).

We also ensured that the ADP-dependent multimerization that is observed at high protein concentration and enhanced by the M96T mutation is of the same nature, i.e. involves the same protomer–protomer interface, as the native inducer-dependent multimerization of MalT. For that purpose, we took advantage of the R171E substitution, which specifically alters the protomer–protomer interface in the native MalT oligomers and thus interferes with MalT multimerization (49). We introduced R171E in the MalT^M96T^ variant, purified MalT^M96T,R171E^ and MalT^R171E^ and analyzed them by size-exclusion chromatography as described above. As expected, MalT^M96T,R171E^ and MalT^R171E^ both behaved as monomers whatever the concentration (Figure 6B), showing that the M96T substitution is not able to enhance multimerization of the MalT^R171E^ variant. Note that neither R171E nor M96T–R171E interfered with the activation pathway upstream from the multimerization step since both MalT^R171E^ and MalT^M96T,R171E^ underwent the same maltotriose-induced conformational changes as MalT, as judged from limited proteolysis assays (Supplementary Figure S11B).

**Figure 7.**
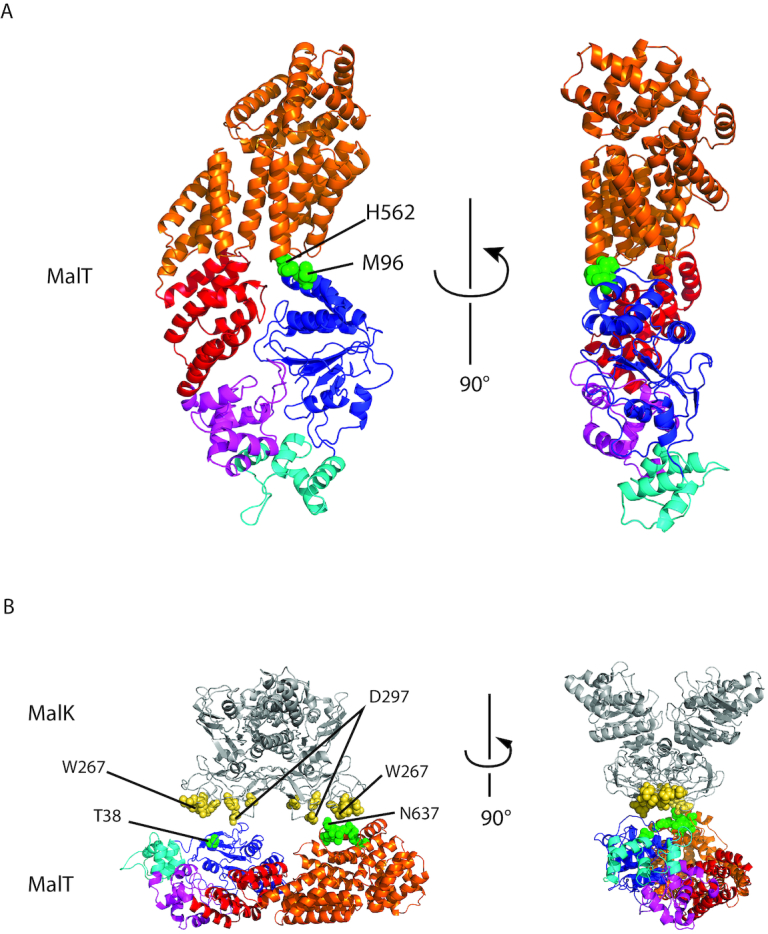
MalT 3D model. MalT was modeled without its DNA-binding domain, using the PH0952ΔN structure and the MalT sensor structure (1HZ4), as described. (**A**) MalT model depicted as cartoon representation with residues M96 and H562 represented by green spheres. (**B**) MalT model manually juxtaposed with MalK structure (PDB code: 3FH6) with patches predicted to be involved in MalT regulation by MalK (MalT: green spheres, MalK: yellow spheres) facing each other.

In conclusion, we have demonstrated here that the MalT protein harbors a sensor–NBD interface, whose disruption displaces the equilibrium between the resting and active form of the protein toward the latter, and which hence participates in the autoinhibition of the protein activity. The knowledge of the existence of this interface allowed us to refine the model of the MalT protein without its DNA-binding domain (Figure 7), which will be helpful for further studies of MalT family bacterial activators and serine-threonine kinases.

## DISCUSSION

### An NBD–sensor interaction strengthens the arm-based autoinhibition of STAND proteins with a TPR sensor

We have solved the crystal structure of PH0952, a putative transcription regulator of *P. horikoshii*, which stands out among archaeal transcription factors by its relatively high molecular weight and multidomain architecture (50). The functionality of the *PH0952* gene is strongly supported by its conservation in the genomes of over 10 Thermococcales species as well as several other members of the phyla Euryarchaeota, Crenarchaeota and lineages of uncultivated archaea, including the recently discovered Marsarchaeota.

**Figure 8.**
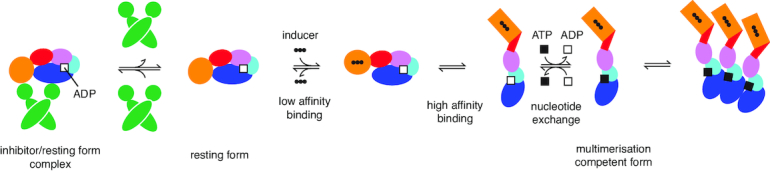
The double autoinhibition mechanism of the MalT STAND archetype. The resting protein is stabilized by inhibitors. In conditions relieving inhibitor action (for MalK, substrate transport through the cytoplasmic membrane), a first binding step involving low-affinity inducer binding coupled to disruption of sensor–NBD interactions is possible. Recruitment of the arm to assemble a high-affinity inducer binding site then releases the arm–NBD contacts which maintained the NOD in the closed conformation (8). Opening of the NOD allows for nucleotide exchange and subsequent multimerization.

PH0952 is the first STAND ATPase harboring a TPR-type sensor whose structure is solved. Interestingly, it reveals that resting PH0952 exhibits NBD–sensor contacts as already observed for APAF1 and NLRC4. By using a structure-guided mutagenesis approach, we have further shown that similar contacts contribute to the autoinhibition of MalT, a well-characterized STAND homolog of PH0952 that also harbors a TPR-type sensor. This NBD–sensor interaction adds to the NBD–arm interaction previously shown to maintain MalT NOD in the closed state (8), which implies that the two-step inducer-binding scenario revealed by the latter study is more complex than anticipated. Indeed, the first, low-affinity inducer binding step is characterized by a hinge motion of the sensor with respect to the arm domain while the latter is still interacting with the NBD (8). If we assume a rigid body movement of the sensor, the first binding step is incompatible with the NBD–sensor contacts identified here. Therefore, disruption of these NBD–sensor contacts is presumably coupled with the low affinity inducer-binding step as disruption of the arm–NBD contacts is coupled with the high affinity inducer-binding step (Figure 8). This is reminiscent of the scenario proposed for APAF1 activation, in which cytochrome c first binds the WD2 lobe of the sensor, probably with low affinity, and the subsequent movement of the WD1 lobe requires breaking the few NBD–WD1 contacts before disruption of the arm–NBD autoinhibitory contacts enable the formation of a higher affinity binding site (9,45).

More generally, STAND activation seems to be a multistep process in which each step consists of the exposure of an inducer-binding determinant coupled to the disruption of a specific set of autoinhibitory contacts, a mechanism expected to increase specificity. This property is probably favored by the spiral shape adopted by these proteins, with the sensor capping the NBD, an architecture that is made possible by the crab-claw shape of the NOD module and might be a reason for the success of the NOD in immunity proteins. The two STANDs, NAIP2 and NAIP5, are a good illustration of the way STAND proteins achieve exquisite specificity, although the exact scenario of their activation remains to be determined. Indeed, NAIP5 binds its inducer flagellin through no less than 6 domains, while its close homolog NAIP2 uses a different set of domains to recognize a different inducer, PrgJ (51).

While NBD–sensor contacts turn out to be a recurrent feature of resting STANDs whatever the sensor structure, the NBD region involved in these seemingly weak interactions is not fully conserved. In APAF1 and in PH0952/MalT, the same ISM region of the NBD is contacted by a WD-40 sensor and a TPR-type sensor, respectively. In contrast, the LRR sensor of NLRC4 contacts a different region of the NBD, namely one of the β-hairpins of the R0 region preceding the conserved NBD core. These differences may reflect different modes of activation of these STAND proteins. NLRC4 is activated allosterically by the binding of an activated inducer-bound NAIP molecule (and then in a prion-like manner, by other activated NLRC4 molecules). This is only possible because one face of NLRC4 (called the receptor surface (52)) is accessible to the opposite face (the catalytic surface (52)) of NAIP. This type of activation has probably co-evolved with autoinhibitory contacts that do not mask the receptor surface of NLRC4. On the contrary, in the case of APAF1, each monomer has to be activated to trigger the formation of the active homomultimer (7,45). In this case and possibly in the case of STAND proteins with a TPR sensor, different autoinhibitory contacts could have arisen, preventing activation by the catalytic surface if exposed by accident (e.g. in the case of a protein with a proteolyzed sensor). An interaction of the sensor with the ISM region, which lies close to the receptor surface is indeed expected to prevent an unwanted NLRC4-like activation.

### The NBD–sensor contact, a key element for stabilization of resting STAND proteins by inhibitors

The model of MalT in the resting form obtained here using information from the PH0952 structure and a cysteine pair mutagenesis strategy (Figure 7A) also sheds light on another mechanism, whereby MalT and other STAND proteins are maintained in the resting form, namely through interaction with inhibitors (53–56). Earlier work showed that MalFGK_2_, the ABC transporter specific for maltodextrins (the substrate of the enzymes encoded by the MalT regulon), inhibits MalT through direct protein–protein interaction in the absence of transport, thus preventing activation of MalT by endogenously produced inducer. The transporter component involved in MalT sequestration is the MalK_2_ ATPase dimer located at the inner face of the cytoplasmic membrane. The MalT–MalK interaction was shown to depend on at least two determinants on MalT, one on the NBD (the T38 patch (56)), the other on the sensor (the N637 patch (37)) and two determinants on the C-terminal domain of MalK (the W267 and D297 patches, (57,58)). Up to now, it was unclear how the MalT and MalK determinants interacted with each other.

Analysis of our structural model of MalT juxtaposed with the structure of MalK in the resting transporter (PDB code: 3FH6 (59)) reveals that the two MalT determinants (i) lie on the same face of the protein, like the four patches of the MalK dimer and (ii) are separated by ∼45–66 Å (depending on the atoms considered), consistent with their interaction with the two MalK W267 patches (span: ∼47–73 Å) or with one protomer W267 patch and the other protomer D297 patch (span: ∼31–55 Å) (Figure 7B). Note that a similar model for the MalK–MalT interface had been proposed earlier even though no structural information was available on MalT (60).

In conclusion, while mutation-induced disruption of the NBD–sensor contacts causes a small effect on MalT activity *in vitro*, these autoinhibitory contacts are expected to play an important role *in vivo* by maintaining the MalT NBD and sensor patches in register with the MalK patches. We presume that inhibitor-enhanced autoinhibitory interactions prevail in the STAND superfamily, given the role often played by these proteins in ‘life or death’ decisions.

## DATA AVAILABILITY

Atomic coordinates and structure factors have been deposited in the protein data bank under the accession code 6MFV.

## Supplementary Material

Supplementary Data
