# Supplementary material for: Double autoinhibition mechanism of signal transduction ATPases with numerous domains (STAND) with a tetratricopeptide repeat sensor
Source: Nucleic Acids Res. 2019 Feb 21;47(7):3795–810. doi: 10.1093/nar/gkz112 (PMC6468293; doi:10.1093/nar/gkz112)
Supplement: Supplementary Data [file gkz112_supplemental_file.pdf]

Supplementary Material and Methods

Plasmids

pOM168 was constructed by cloning a PCR fragment containing the sequence encoding residues 102-753 of *PH0952* amplified from the *Pyrococcus horikoshii* OT3 chromosome between the XbaI and KpnI sites of pKYB1, so as to yield the following sequence between these two sites.

```
 XbaI                                  NcoI                                     KpnI
TCTAGAAATAATTTTGTTTAACTTTAAGAAGGAGATATACCATGGCCCCT-PH0952-AACTTTAAGTGCTTTGCCAAGGGTACC
                                          M  A  P ........ N  F  K  C  F  A  K  G  T
                                            102                 753 1  2  3  4  5  6
                                             <   PH0952 sequence  >< Sce VMA1 intein gene
                                                                         sequence
```

pOM258 is a derivative of pOM257 (16): like this plasmid it contains a $P_{KAB\text{-}TTGG}$-*malT* fragment cloned between the EcoRI and BamHI sites of pJM241, but without the silent SacI site at +21 of the *malT* sequence and with a KpnI silent site at +2478.

pOM260 is a derivative of pOM215 (60) which contains a $P_{KAB\text{-}TTCT}$-*malT* fragment cloned between the EcoRI and BamHI sites of pJM241 without the SacI silent site at 2381 but with a KpnI silent site at +2478 which makes the sequence of the *malT* gene identical to that of pOM258.

Growth media

Growth media for purification of PH0952ΔN and selenomethionine substituted PH0952ΔN contained 100 µg/mL kanamycine and 10 µg/mL chloramphenicol.

The medium used for production of the selenomethionine substituted PH0952ΔN contained 50 mM Na_2HPO_4, 50mM KH_2PO_4, 25 mM $(NH_4)_2SO_4$, 2 mM $MgSO_4$, 0.2x trace metals (19), 0.5% glucose, 10 µg/mL L-methionine, 100 µg/mL L-selenomethionine, 1.5 mM of 17 amino-acids (no C, M, Y), 100 nM vitamin B12 and 1 mM IPTG.

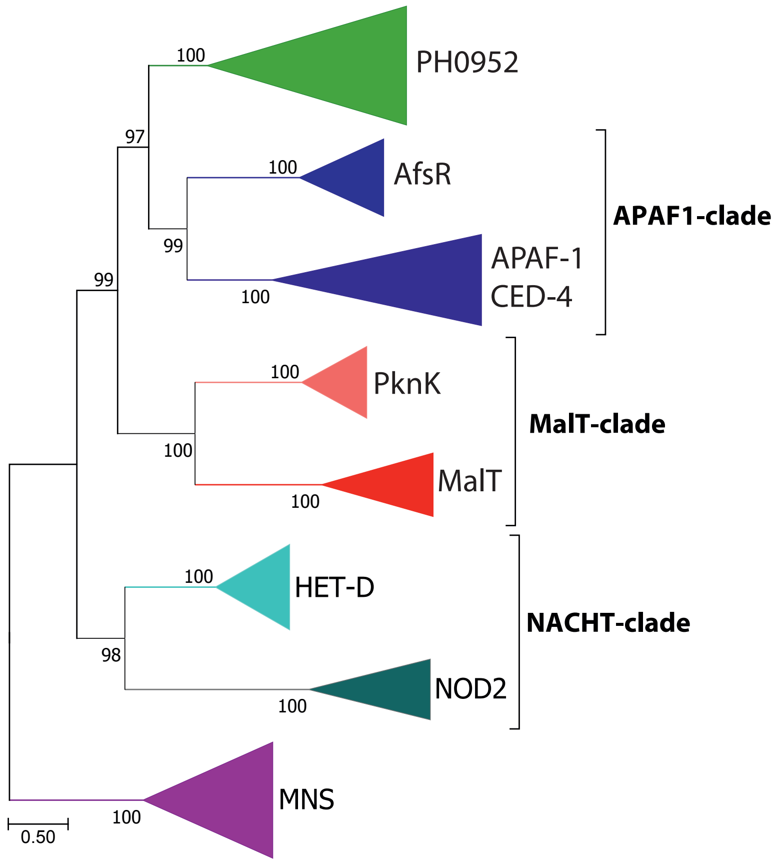

Figure S1: Maximum likelihood phylogenetic analysis of STAND ATPases. The tree contains representative members from different families of STAND proteins. Numbers at the branch points represent the Bayesian-like transformation of aLRT (aBayes) local support values. The scale bar represents the number of substitutions per site. The tree is rooted in the branch leading to the MNS clade (1). Searches against expanded genomic databases indicate that PH0952-like ATPases are more broadly distributed in archaeal lineages than anticipated, particularly in Euryarchaeota, but also in members of the phylum Crenarchaeota and various recently discovered uncultivated archaea. Contrary to previous hypotheses of a horizontal transfer of *PH0952* into archaea from bacteria or eukaryotes (1,38), our analysis (see also Fig. 1C) suggests that PH0952-like ATPases do not fall into any of the other previously established families of STAND ATPases, but rather represent a distinct family within the STAND superfamily.

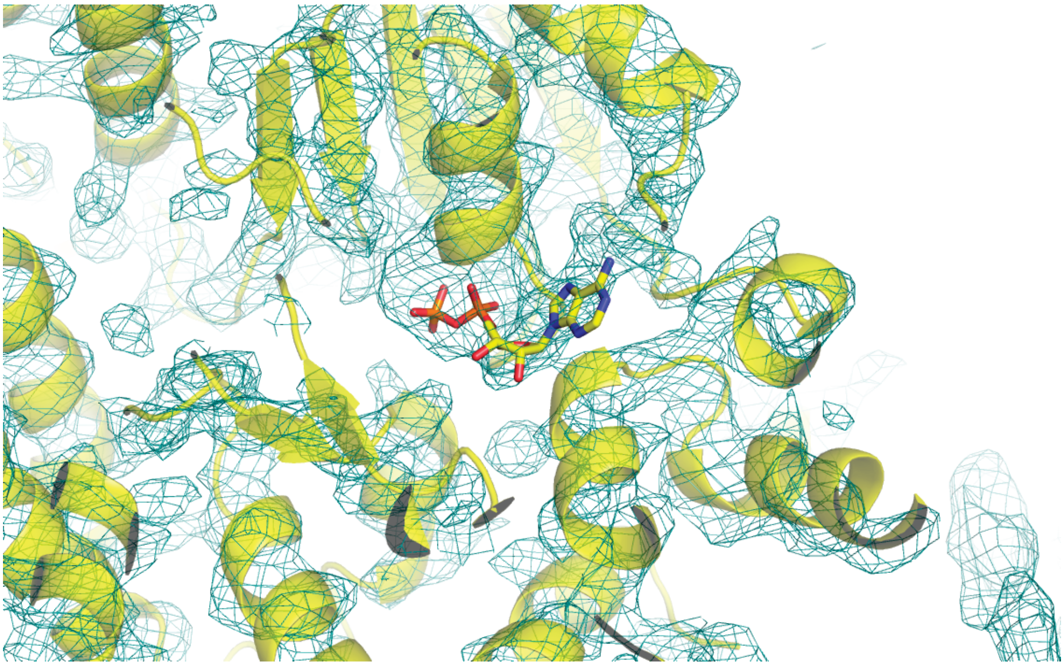

Figure S2: The ADP binding site of PH0952. The protein is shown in cartoon representation and the ADP molecule is depicted in sticks. The original SAD-phased, density modified electron density map (blue mesh) is contoured at 1.5 σ.

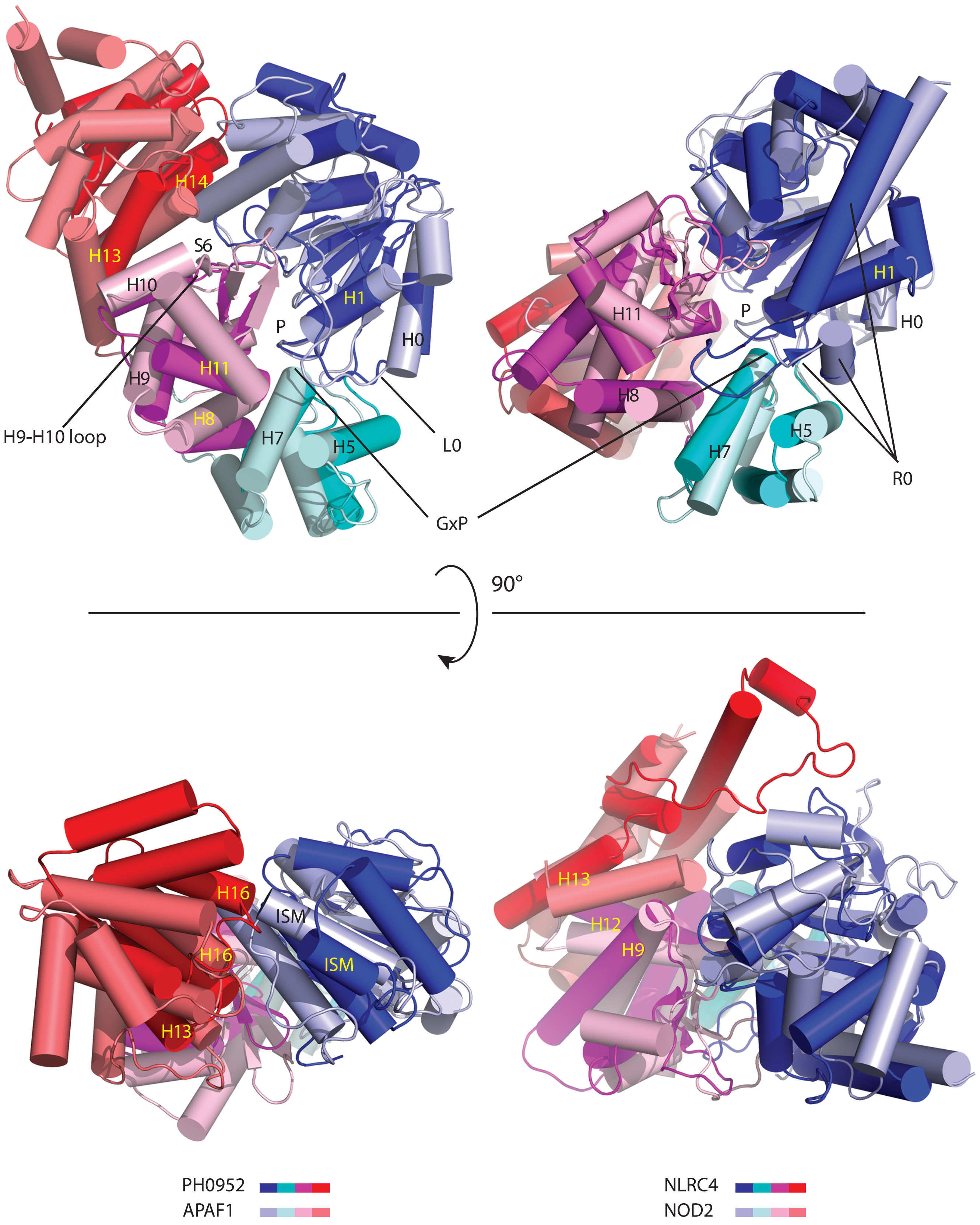

Figure S3: Interactions between consecutive domains in the NOD-arm module of STAND proteins. The NOD-arm regions of PH0952/APAF1 (left) and NLRC4/NOD2 (right) were superimposed through their P-loop regions and put side by side in the same orientation, in two perpendicular views (top and bottom). Relevant secondary structure elements are indicated, either once when the superimposition is good, or twice when the two structures do not overlap.

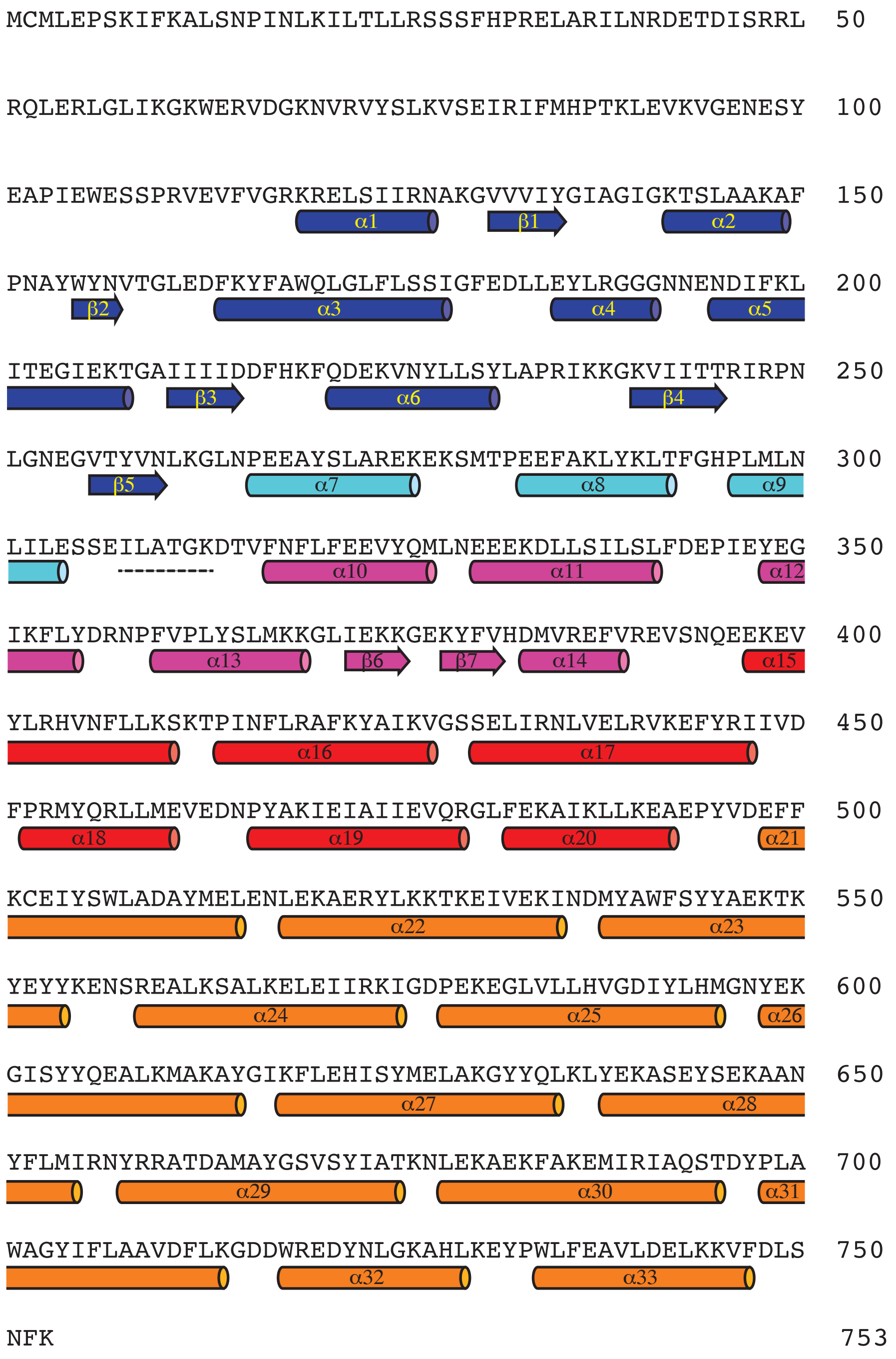

Fig. S4 : Secondary structure of the PH0952 derived from the atomic coordinates determined in this work. The colour code for domains is as in Fig. 1. The dashed line indicates the unresolved region between the HD and the WHD.

No:	Chain	Z	rmsd	lali	nres	%id	PDB	Description
1:	3q78-A	7.3	3.2	90	312	10	PDB	MOLECULE: FARNESYLTRANSFERASE ALPHA SUBUNIT;
2:	5jz6-A	7.2	3.4	89	429	15	PDB	MOLECULE: ASPARTYL/ASPARAGINYL BETA-HYDROXYLASE;
3:	5jjt-B	7.2	3.1	90	479	7	PDB	MOLECULE: SERINE/THREONINE-PROTEIN PHOSPHATASE 5;
4:	3sfy-A	7.1	3.2	90	315	10	PDB	MOLECULE: CRYPTOCOCCUS NEOFORMANS PROTEIN FARNESYLTRANSFERA
5:	3q79-A	7.1	3.3	90	314	10	PDB	MOLECULE: FARNESYLTRANSFERASE ALPHA SUBUNIT;
6:	3q75-A	7.1	3.2	90	315	10	PDB	MOLECULE: FARNESYLTRANSFERASE ALPHA SUBUNIT;
7:	1n4s-C	7.1	3.1	89	314	11	PDB	MOLECULE: PROTEIN FARNESYLTRANSFERASE/GERANYLGERANYLTRANSFE
8:	3q73-A	7.1	3.3	90	316	10	PDB	MOLECULE: FARNESYLTRANSFERASE, ALPHA SUBUNIT;
9:	3q7f-A	7.1	3.3	90	313	10	PDB	MOLECULE: FARNESYLTRANSFERASE ALPHA SUBUNIT;
10:	5jjt-A	7.0	3.1	88	471	7	PDB	MOLECULE: SERINE/THREONINE-PROTEIN PHOSPHATASE 5;

Fig. S5 : First 10 structural homologs found by a Dali search (october 24, 2017) seeded with the coordinates of the PH0952 arm. Headers in red indicate TPR proteins. The other proteins retrieved have a farnesyl-transferase α-subunit fold, which differs from TPR repeats essentially by the twist (i.e. the way consecutive two-helix bundles are spatially related to each other, (44))

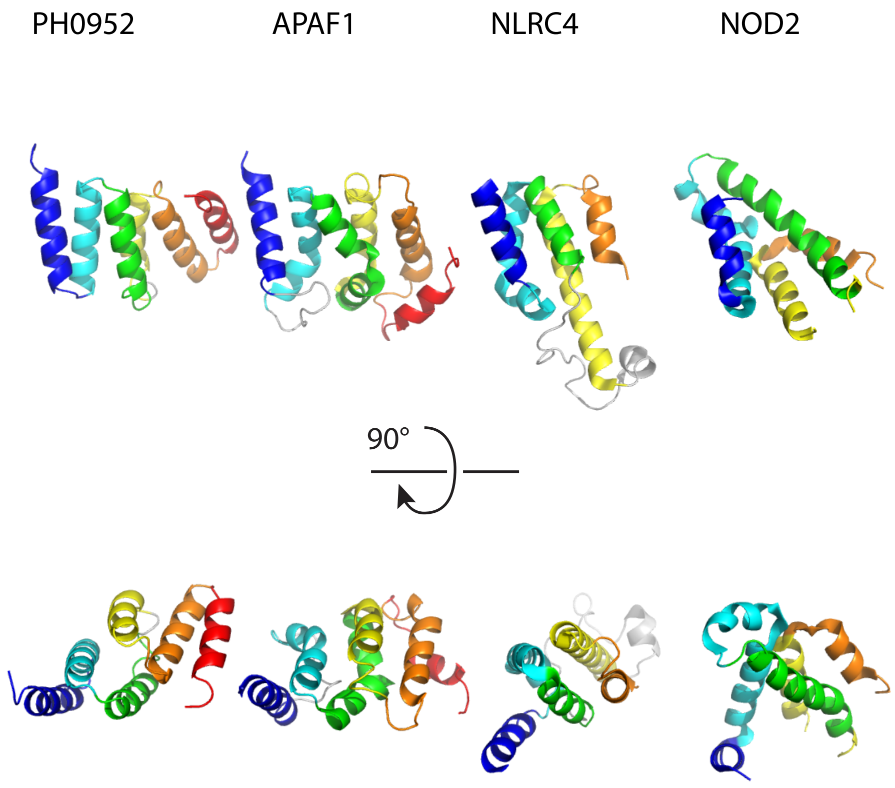

Fig. S6: Comparison of the 4 STAND arm domains for which a crystal structure is available. Helices are rainbow colored from blue (first helix) to red (6th helix). Possible insertions are represented in grey.

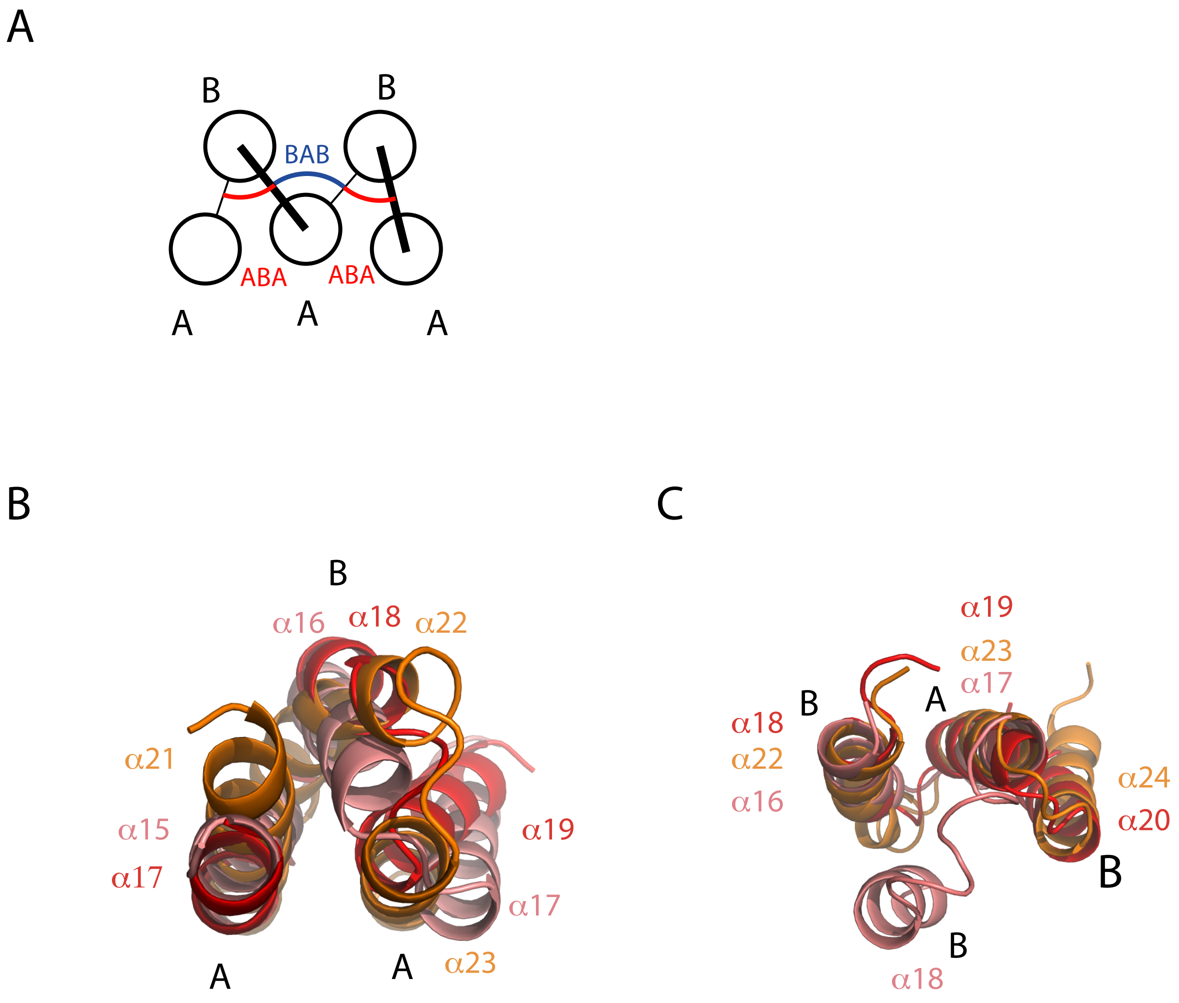

Fig. S7: A. Schematic representation of a typical TPR fold wiewed along the helix longitudinal axis (helices are represented by circles and turns by dashes). ABA (red) and BAB (blue) angles are shown. B,C. Superimposition of groups of three consecutive helices from the arm (α15 to α20) and as a comparison, from the TPR sensor domain of PH0952 (α21 to α24). B. ABA angles. C. BAB angles.

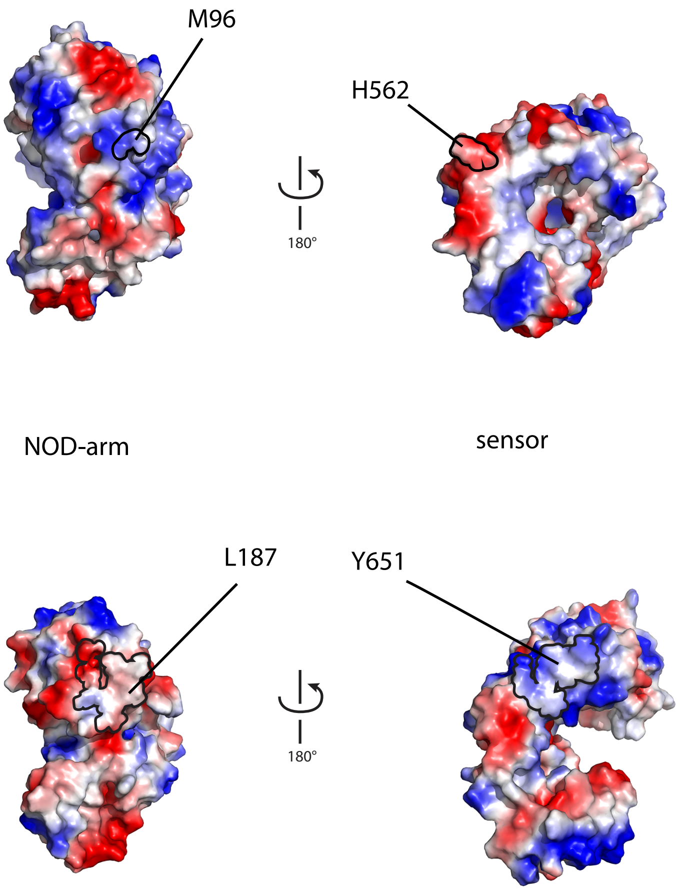

Fig. S8: Opposite charge complementarities in the MalT homology model (top) and in the PH0952 X-ray structure (bottom). Electrostatic potential surfaces are shown after splitting the structure in two at the arm sensor junction. The PH0952 sensor-NBD interface patches are contoured and a representative residue is indicated.

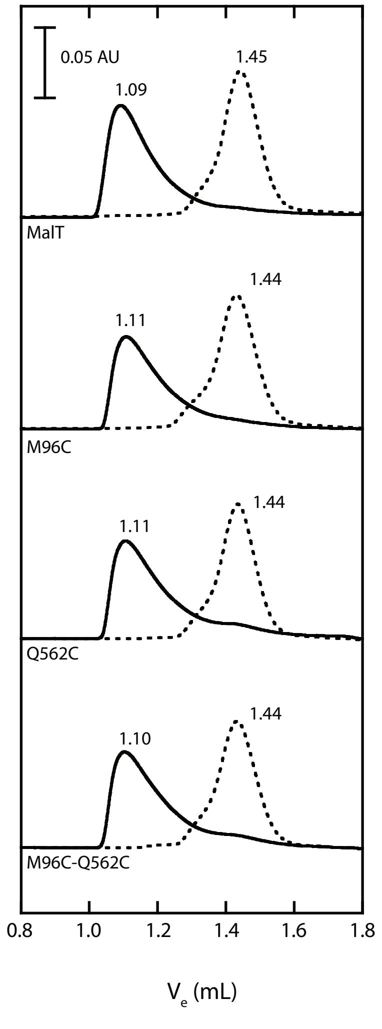

Fig. S9: The cysteine mutations do not affect MalT activation. $MalT^{M96C}$, $MalT^{H562C}$ and $MalT^{M96C\text{-}H562C}$ display inducer dependent multimerization as MalT in reducing conditions. Proteins (10 µM) were preincubated for 30 min in a Tris-HCl buffer (50 mM, pH 8.0) containing 10% sucrose, 0.1 M KCl, 0.033 M KI, 0.017 mM K_3 citrate,10 mM Mg acetate, 0.1 mM EDTA, 2 mM dithiothreitol, 0.18 mM ATP. They were injected on a superdex 200 column equilibrated with a Tris-HCl buffer (50 mM, pH 8.0) containing 0.033 M K_3 citrate, 10 mM Mg acetate, 0.1 mM EDTA, 1 mM dithiothreitol, 0.1 mM ATP. Preincubation and running buffer contained maltotriose 1 mM only in the experiments displayed by solid curves.

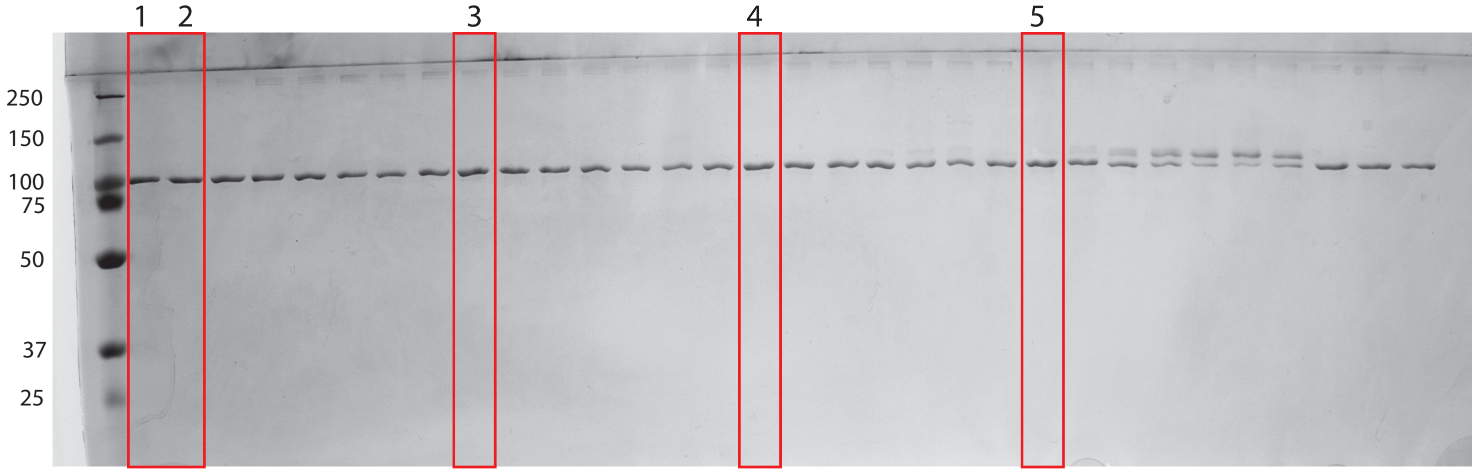

Fig. S10: MalT, MalT^{M96C}, MalT^{H562C} and MalT$^{M96C\text{-}H562C}$ all migrate at the same level in the presence of DTT. The gel used to generate Fig. 5a is shown in its entirety to enable comparison of the migrations of the four different proteins in reducing conditions. +DTT lanes are highlighted for clarity. 1, 2 : MalT, 3: MalT^{M96C}, 4: MalT^{H562C},5: MalT^{M96C,H562C}. Molecular weights of the markers are indicated in kDa.

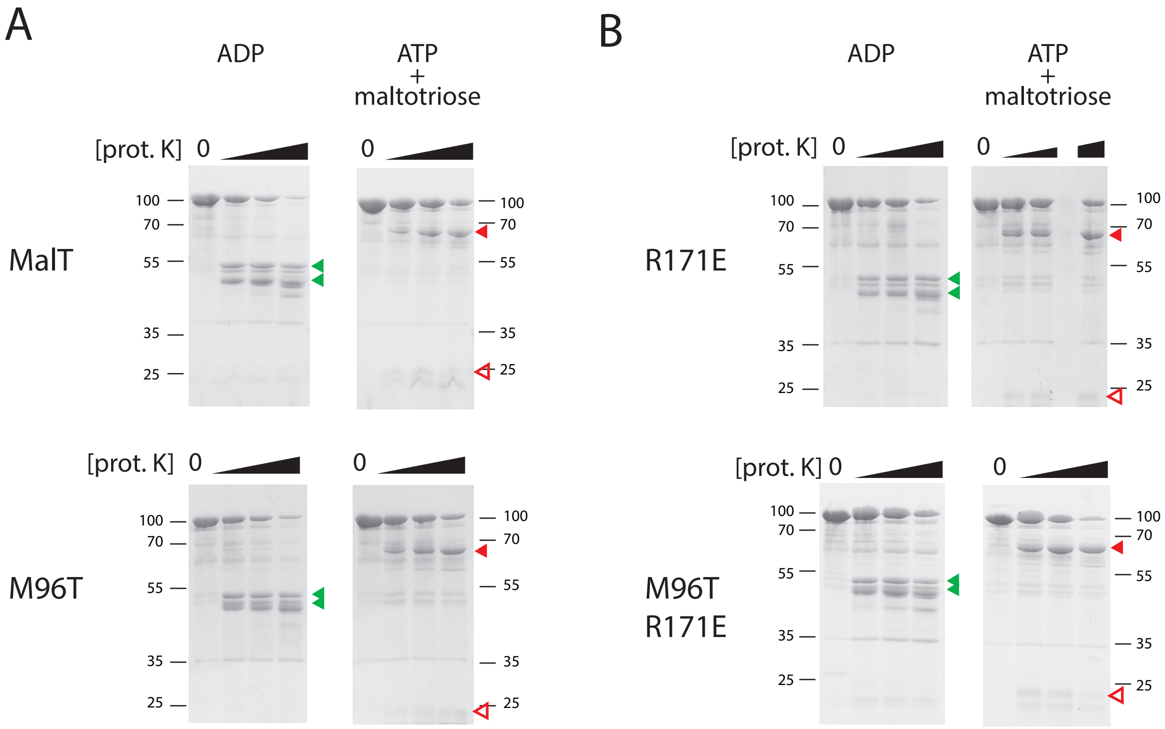

Fig. S11: The *M96T* mutation does not interfere with the maltotriose-induced conformational changes undergone by MalT or MalT^{R171E}. Limited proteolysis of MalT and variants by proteinase K in ADP and ATP + maltotriose. The same increasing K/MalT (w/w) ratios (0, 1:533, 1:267, 1:133) were used for all eight experiments. Green arrows point at the 50 kDa and 45-48 kDa fragments specific of the resting form. Red arrows points at the 66 kDa and 25 kDa fragments specific of the activated form. A. MalT, MalT^{M96T}. B. MalT^{R171E}, MalT^{M96T,R171E}.

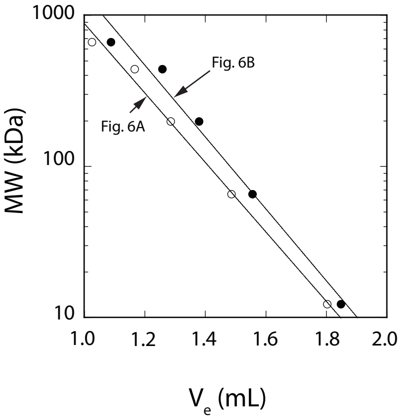

Fig. S12: Calibration curves for the columns used in the experiments of Fig. 6A (open circles) and in those of Fig. 6B (closed circles). Markers (thyroglobulin, 669 kDa, apoferritin, 443 kDa, amylase, 200kDa, bovine serum albumin, 66 kDa, cytochrome C, 12.4 kDa) were injected on the columns equilibrated in Tris-HCl buffer (50 mM, pH 8.0) containing 10% sucrose, 0.033 M K3 citrate, 10 mM Mg acetate, 0.1 mM EDTA, 1 mM dithiothreitol and 0.1 mM ADP. Monomeric MalT-ADP run at 10 µM on the Fig. 6B column eluted at 1.484 mL. Theoretical elution volumes for a globular protein of the same molecular weight as MalT calculated from the calibration curves are 1.40 mL (Fig. 6A column) and 1.47 mL (Fig. 6B column).
